# A mathematical model for assessing the effectiveness of controlling relapse in *Plasmodium vivax* malaria endemic in the Republic of Korea

**DOI:** 10.1371/journal.pone.0227919

**Published:** 2020-01-24

**Authors:** Sungchan Kim, Jong Hyuk Byun, Anna Park, Il Hyo Jung

**Affiliations:** 1 Department of Mathematics, Pusan National University, Geumjeong-Gu, Busan 46241, Republic of Korea; 2 Finance · Fishery · Manufacture Industrial Mathematics Center on Big Data, Pusan National University, Geumjeong-Gu, Busan 46241, Republic of Korea; Faculty of Science, Ain Shams University (ASU), EGYPT

## Abstract

Malaria has persisted as an endemic near the Demilitarized Zone in the Republic of Korea since the re-emergence of *Plasmodium vivax* malaria in 1993. The number of patients affected by malaria has increased recently despite many controls tools, one of the reasons behind which is the relapse of malaria via liver hypnozoites. *Tafenoquine*, a new drug approved by the United States Food and Drug Administration in 2018, is expected to reduce the rate of relapse of malaria hypnozoites and thereby decrease the prevalence of malaria among the population. In this work, we have developed a new transmission model for *Plasmodium vivax* that takes into account a more realistic intrinsic distribution from existing literature to quantify the current values of relapse parameters and to evaluate the effectiveness of the anti-relapse therapy. The model is especially suitable for estimating parameters near the Demilitarized Zone in Korea, in which the disease follows a distinguishable seasonality. Results were shown that radical cure could significantly reduce the prevalence level of malaria. However, eradication would still take a long time (over 10 years) even if the high-level treatment were to persist. In addition, considering that the vector’s behavior is manipulated by the malaria parasite, relapse repression through vector control at the current level may result in a negative effect in containing the disease. We conclude that the use of effective drugs should be considered together with the increased level of the vector control to reduce malaria prevalence.

## Introduction

Malaria has persisted in the Republic of Korea since the 1993 *Plasmodium vivax* (*P. vivax*) malaria re-emergence [1]. According to KCDC (Korea Centers for Disease Control & Prevention), the Korean Government Health Authorities’ continued efforts had managed to reduce the number of malaria patients to a few hundreds, but recently the number has started to increase again.

Globally, *P. vivax* malaria was one of the most neglected diseases in the world due to its low rate of fatality, but recently there has been much interest ever since it has been found that severe symptoms and deaths due to vivax malaria are not uncommon [2], even in Korea [3]. In particular, *P. vivax* is known to produce hypnozoites, which are dormant forms of the parasite residing in the liver of the victim [4]. Activation of these hypnozoites makes future relapse possible even if blood-stage malaria is removed from primary infection. Hypnozoites are produced when the conditions for transmission of the parasite are less favorable, for instance, during short seasons of appropriate temperatures and vectors available at high latitudes [5]. The relapse rate caused by these hypnozoites is around 68% across the world, without any popular treatment for such relapses [6]. Therefore, treatment to prevent malaria relapse by removing the remaining hypnozoites in the liver is imperative alongside blood-stage malaria treatment.

Treatment of *P. vivax* malaria in Korea generally uses *Chloroquine* for schizonticide in the blood and *Primaquine* for detoxification in the liver, termed ‘radical cure’ [7]. Until now, *Primaquine* has been the only drug used for radical cure. *Primaquine* has a short half-life, so patients faced considerable difficulty in taking their daily medications during the recommended period of 14 days during which dormant hypnozoites in the liver were treated [8]. Thus, the misuse of drugs in patients treated with blood-stage malaria often resulted in poor treatment rate for hypnozoites [8]. However, with *Tafenoquine* being approved as a new drug in relapse treatment by the U.S. FDA in 2018. *Tafenoquine* only requires a single dose to prevent relapse of malaria due to its long half-life of about 16 days [9]. The development of this therapy reduces the effort required from an individual during his treatment. Therefore, proper use of *Tafenoquine* in addition to the use of *Primaquine* may lower the relapse rate even at the current level [10].

Meanwhile, a model for evaluating malaria relapse patterns has been introduced by Roy et al. [11]. In this model, the authors developed a mathematical model that uses gamma-distributed lag-time to cover the mosquito data and the absence of relapse dynamics, demonstrating a new technique to estimate the rate of relapse of infections in northwest India. In addition, based on the estimated values of the parameters, the effect of anti-relapse on the area was evaluated. However, the model presented above is not suitable for use in our research because, unlike northwest India, the northernmost part of Korea has a yearly variation in temperature of more than 30°C and is not a mosquito-friendly environment between November and February. Additionally, due to the higher latitudes of the region, the distribution of the period between the initial infection and the first relapse has to be expressed in a multimodal form. Furthermore, the distribution of latent period of malaria in humans is expressed in short-term and long-term bimodal forms. The Gamma distributed model—a generalized exponential model—has been widely used for modeling realistic distributions that the exponentially distributed model cannot be used for [12–16]. However, it was revealed that the exponential or gamma distributions, which are commonly used to model the incubation and relapse periods, are inadequate in capturing the complexity of the event-time distribution of *P. vivax* malaria infections in the temperate regions being considered [5]. Therefore, for this study, a transmission model that considers multimodal distributions is essential. In this paper, we develop a transmission model to evaluate the rate of malaria relapse infections in the northern part of Korea and to examine its effect at the population-level on radical cure. This model takes into account data regarding the mosquito population of the region and the intrinsic distribution of relapses based on existing literature on malaria models in Korea [17–19]. To model intrinsic distributions of incubation periods and the time between the initial infection and the first relapse, we introduce a Coxian distribution, which can express multimodal phenomena. Our model measures the effectiveness of the new mass treatment for relapse at the population-level.

## Materials and methods

### Data

#### Incidence in targeted area

In this study, we focus on the outbreak of *P. vivax* malaria in Yeoncheon-gun, Gyeonggi-do in Korea in 2007. Yeoncheon-gun is one of the places in Korea that suffers from the most severe malarial endemic. Geographically, it is located near the Demilitarized Zone (DMZ). *Anopheles sinensis* is the most popular malaria-bearing mosquito in the region. We used weekly malaria incidence data from the Infectious Disease Portal of Korea Centers for Disease Control & Prevention (KCDC). This region has four distinct seasons with average temperatures in summer and in winter. According to Korea Meteorological Administration (KMA), the lowest average monthly temperature is -7.0°C in January, and the highest average monthly temperature is 23°C in August. However, the rainfall is heavy enough to reach 1,300 mm, which is concentrated mostly in summer. Therefore, mosquitoes thrive during the summer, but their population is significantly reduced in winter. Thus, malaria in humans is most prevalent during summer, especially in July and August, and conversely, is least prevalent in winter, especially in December and February.

#### Incubation periods and the time to first relapse

First, a study containing incubation period (IP) data for a total of 229 individuals was identified [20]. This study provides analysis of the data of the incubation periods from cases of civilians infected with *P. vivax* malaria in Korea using data from the malaria epidemiological survey conducted between 2001 to 2010. As malaria in Korea is mainly concentrated in the northwestern region, near Yeoncheon-gun, it is reasonable to use malaria incubation data of the entire country for this study. Data points were recorded from the histogram presented in the study. Next, the time to first relapse (TTFR) data was obtained from [21]. This paper presents raw TTFR data for 46 relapsed samples from South and North Korea.

**Fig 1:**
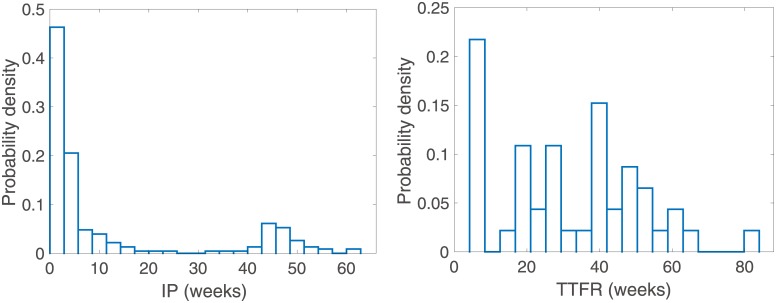
Normalized histogram of incubation periods between 2001 and 2010 (left), and time to first relapse (right) in Korea.

Fig 1 depicts the histograms indicating the relative frequencies of IPs and TTFRs for *P. vivax* malaria in human. The distribution of the histogram clearly explains why the malaria parasite survives, although mosquitoes cannot survive in the region during winter. Both incubation and relapse occur in two forms—short-term and long-term. Malaria parasites undergo short-term incubations of about 1-2 weeks and long-term incubations of about a year [22], and relapse due to dormant hypnozoites in the liver occurs about 8-10 weeks after the primary attack in the short-term and about a year afterwards in the long-term, depending on the specific strain of virus [23, 24]. The spread of the disease during the succeeding year is also affected by this.

### Mathematical model

**Fig 2:**
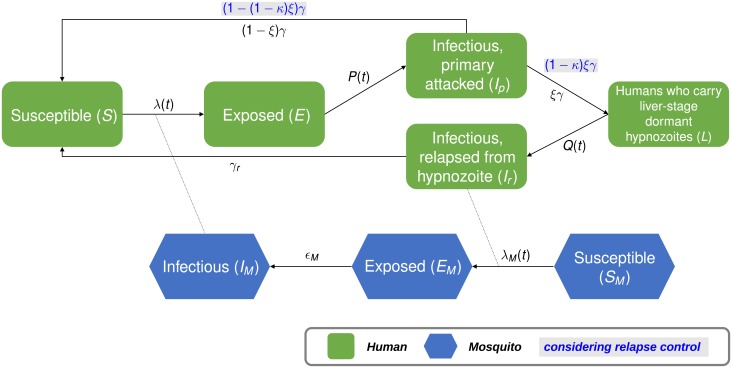
Schematic diagram of the model (1). Human classes are represented as green rectangles with rounded corners, and mosquito classes as elongated blue hexagons. Arrows indicate the directions of transition between classes and dotted lines indicate the relationships between actions.

We formulate a mathematical model to capture the trend of relapse of *P. vivax* malaria at the population-level, which has been schematically depicted in Fig 2:

$$\begin{array}{c} \begin{array}{cc} \frac{\text{d}S(t)}{\text{d}t} & =\mu -\lambda \left(t\right)S\left(t\right)+\left(1-\left(1-\kappa \right)\xi \right)\gamma I_{p}\left(t\right)+\gamma_{r}I_{r}\left(t\right)-\mu S\left(t\right),\\ E(t) & =\int_{0}^{t}\lambda \left(t-u\right)S\left(t-u\right)\;\text{exp}\left(-\mu u\right)P\left(u\right)\;\text{d}u,\\ \frac{\text{d}I_{p}\left(t\right)}{\text{d}t} & =\int_{0}^{t}\lambda \left(t-u\right)S\left(t-u\right)[-P_{u}\left(u\right)]\;\text{exp}\left(-\mu u\right)\;\text{d}u-\left(\gamma +\mu \right)I_{p}\left(t\right),\\ L(t) & =\int_{0}^{t}\left(1-\kappa \right)\xi \gamma I_{p}\left(t-u\right)Q\left(u\right)\;\text{exp}\left(-\mu u\right)\;\text{d}u,\\ \frac{\text{d}I_{r}\left(t\right)}{\text{d}t} & =\int_{0}^{t}\left(1-\kappa \right)\xi \gamma I_{p}\left(t-u\right)[-Q_{u}\left(u\right)]\;\text{exp}\left(-\mu u\right)\;\text{d}u-\left(\gamma_{r}+\mu \right)I_{r}\left(t\right),\\ \frac{\text{d}S_{M}\left(t\right)}{\text{d}t} & =\mu_{M}-\lambda_{M}\left(t\right)S_{M}\left(t\right)-\mu_{M}S_{M}\left(t\right),\\ \frac{\text{d}E_{M}\left(t\right)}{\text{d}t} & =\lambda_{M}\left(t\right)S_{M}\left(t\right)-\left(\epsilon_{M}+\mu_{M}\right)E_{M}\left(t\right),\\ \frac{\text{d}I_{M}\left(t\right)}{\text{d}t} & =\epsilon_{M}E_{M}\left(t\right)-\mu_{M}I_{M}\left(t\right), \end{array} \end{array}$$ (1)

where *ρ*(*t*) = *M*(*t*)/*H*, λ(*t*) = *bp*_*H*_ *ρ*(*t*)*I*_*M*_(*t*), and λ_*M*_(*t*) = *bp*_*M*_(*I*_*p*_(*t*) + *I*_*r*_(*t*)).

In the model (1), the component related to humans is formulated by dividing them into five classes (Table 1): *S*, *E*, *I*_*p*_, *L* and *I*_*r*_, which describe the groups of susceptible, exposed, primary infected, humans who carry liver-stage dormant hypnozoites, and infectious individuals who have relapsed from the re-emergence of hypnozoites. The population *M* of female *Anopheles* mosquitoes is divided into three classes: *S*_*M*_, standing for susceptible vectors, *E*_*M*_, standing for exposed vectors, and *I*_*M*_, standing for infectious vectors. The total rate in each case remains constant, i.e., *S*(*t*) + *E*(*t*) + *I*_*p*_(*t*) + *L*(*t*) + *I*_*r*_(*t*) = 1 and *S*_*M*_(*t*) + *E*_*M*_(*t*) + *I*_*M*_(*t*) = 1. The ratio of the number of mosquitoes to that of humans, *ρ*, represents the seasonality of transmission, and therefore *ρ* ≡ *ρ*(*t*). As demonstrated in the previous section, one of the most important considerations in modeling *P. vivax* malaria in temperate regions is expressing the multimodal nature of IP and TTFR. Therefore, we consider *P*(*t*) and *Q*(*t*), which are the survival functions of the incubation period and the duration of the overall liver stage, TTFR, respectively, to reflect realistic distributions.

**Table 1:** Description of states in model (1).

State	Description
*S*	The fraction of susceptible humans
*E*	The fraction exposed humans
*I*_*p*_	The fraction infectious humans who is primary attected
*I*_*r*_	The fraction infectious humans who is relapsed from hypnozoite first
*L*	The fraction of humans who carry liver-stage dormant hypnozoites
*S*_*M*_	The fraction of susceptible mosquitoes
*E*_*M*_	The fraction of exposed mosquitoes
*I*_*M*_	The fraction of infectious mosquitoes

When a malaria-infected mosquito bites a susceptible human, he/she becomes infected at a rate of λ(*t*) and undergoes an incubation period, which follows the survival probability, *P*(*t*) in the body. Following that, he/she becomes infectious and, with subsequent treatment, leaves the *I*_*p*_-class at the rate of *γ*. One section of this population enters the *L*-class at the rate of *ξ*, as the treatment of hypnozoites in the liver is still not complete, and the other section enter the *S*-class again at the rate of 1 − *ξ* after successful completion of treatment. The period that each individual spends in the *L*-class until the re-emergence of malaria follows the survival probability *Q*(*t*). The relapsed malaria patient in the *I*_*r*_-class then begins to infect susceptible mosquitoes with a force of infection of λ_*M*_(*t*). Relapses can occur for three main reasons: re-infection from a mosquito, re-emergence from untreated hypnozoites in the liver, and from untreated blood-stage malaria. However, it is known that blood-stage malaria has a short treatment period and a high rate of treatment. Therefore, the last reason can be deemed to be statistically insignificant in our case, and we only consider re-infections caused by mosquitoes and untreated hypnozoites. Detailed descriptions of relevant parameters are recorded in Table 2.

**Table 2:** Description of parameters.

Parameter	Description	Dimension
*μ*	Per capita rate of birth and death in human	time^−1^
*μ*_*M*_	Per capita rate of birth of adult female *Anopheles* mosquito	time^−1^
1/*γ*	Mean infectiousness period of infectious human who is primary attacked	time
1/*γ*_*r*_	Mean infectiousness period of infectious human who is relapsed	time
*ξ*	Rate of remaining hypnozoites after infectious in the liver at the current level	1
*Q*(*t*)	Survival function of the time-to-relapse (primary infection to the first relapse) are of human at time *t*	1
*κ*	Relapse treatment coverage rate from some interventions	1
1/*ϵ*_*M*_	Mean incubation time of mosquito	time
*P*(*t*)	Survival function of incubation time of human at time *t*	1
*b*	The number of biting human per a mosquito per unit time (=(the number of biting per a mosquito per unit time) × (HBI))	1
*p*_*H*_	The probability of an human infection occur when he/she is bitten by an infected mosquito	1
*ρ*(*t*)	The rate of the number of mosquitoes to the number of humans at time *t*	1
λ(*t*)	Force of infections from mosquito to human at time *t* (= *bp*_*H*_ *ρ*(*t*)*I*_*M*_(*t*))	
*p*_*M*_	The probability of an mosquito infection occur when it bites an infected human	1
λ_*M*_(*t*)	Force of infections from human to mosquito at time *t* (= *bp*_*M*_(*I*_*p*_(*t*) + *I*_*r*_(*t*))	

We consider the control parameter *κ* to observe the effect of group relapse prevention control, which prevents relapse due to hypnozoites. We assume that control at a rate *κ* reduces *I*_*p*_-to-*L* transmission rate *ξ* by (1 − *κ*)*ξ*. Therefore, control at a rate 100 × *κ*% enables individuals from the *I*_*p*_-class to enter *L*-class at a rate (1 − *κ*)*ξγ*, and to enter the *S*-class at a rate (1 − (1 − *κ*)*ξ*)*γ*. Note that *κ* = 0 represents the current level of radical cure, and *κ* = 1 represents the successful control of all primary infected patients occurs.

In addition, we introduce our chosen functions *P* and *Q* as the survival functions of the Coxian distribution. The Coxian distribution, one of the phase-type distributions, is chosen because of its property that any type of non-negative distributions can be approximated to a Coxian distribution [25]. The detailed derivation of the Coxian distributed model is as follows: Let *X* and *Y* be random variables representing the dwelling-times in the exposed stage(*E*) and stage of carrying dormant hypnozoites in the liver (*L*) in humans, respectively. Then the probability density functions of *X* and *Y*, *f*_*X*_ and *f*_*Y*_, respectively, are defined to be

$$\begin{array}{c} f_{X}\left(t\right)=p_{n}\;\text{exp}\left(t\Phi \right)q_{P}\;\;\;\text{and}\;\;\;f_{Y}\left(t\right)=p_{m}\;\text{exp}\left(t\Psi \right)q_{Q}, \end{array}$$ (2)

where

$$\begin{array}{c} \begin{array}{cc} p_{n} & =[1\;\;\;\underset{n-1}{\underset{︸}{0\;\;\;\cdots \;\;\;0}}]\in M_{1\times n}\left(R\right),\\ \Phi & =[\begin{array}{cccccc} -\zeta_{n} & {\bar{p}}_{n-1}\zeta_{n} & 0 & & \cdots & 0\\ 0 & -\zeta_{n-1} & {\bar{p}}_{n-2}\zeta_{n-1} & 0 & \cdots & 0\\ & & & \vdots & & \\ 0 & \cdots & & & -\zeta_{2} & {\bar{p}}_{1}\zeta_{2}\\ 0 & \cdots & & & 0 & -\zeta_{1} \end{array}]\in M_{n\times n}\left(R\right),\\ \Psi & =[\begin{array}{cccccc} -\eta_{m} & {\bar{q}}_{m-1}\eta_{m} & 0 & & \cdots & 0\\ 0 & -\eta_{m-1} & {\bar{q}}_{m-2}\eta_{m-1} & 0 & \cdots & 0\\ & & & \vdots & & \\ 0 & \cdots & & & -\eta_{2} & {\bar{q}}_{1}\eta_{2}\\ 0 & \cdots & & & 0 & -\eta_{1} \end{array}]\in M_{m\times m}\left(R\right),\\ q_{P} & =-\Phi 1_{n}={\left[\begin{array}{ccccc} p_{n-1}\zeta_{n} & p_{n-2}\zeta_{n-1} & \cdots & p_{1}\zeta_{2} & \zeta_{1} \end{array}\right]}^{⊤}\in M_{n\times 1}\left(R\right),\\ q_{Q} & =-\Psi 1_{m}={\left[\begin{array}{ccccc} q_{m-1}\eta_{n} & q_{m-2}\eta_{m-1} & \cdots & q_{1}\eta_{2} & \eta_{1} \end{array}\right]}^{⊤}\in M_{m\times 1}\left(R\right), \end{array} \end{array}$$ (3)

and **1**_*n*_ is an *n* × 1 vector of ones and **Φ** and **Ψ** are transition rate matrices of *X* and *Y*, respectively, where ${\bar{p}}_{i}=1-p_{i}$ and ${\bar{q}}_{j}=1-q_{j}$ for *i* = 1, 2, ⋯, *n* and *j* = 1, 2, ⋯, *m* [26]. Corresponding survival function, *P* and *Q*, are given by

$$\begin{array}{c} \begin{array}{cc} P(t) & =p_{n}\;\text{exp}\left(t\Phi \right)1_{n},\text{and}\\ Q(t) & =p_{m}\;\text{exp}\left(t\Psi \right)1_{m}. \end{array} \end{array}$$ (4)

We successfully derived the Ordinary Differential Equation model from (1) in the case in which *P* and *Q* are assumed to correspond to Coxian distributions (4). The techniques for derivation are as follows: we put the 1 × *n* vector *φ* as

$$\begin{array}{c} \varphi \left(t\right)=p_{P}\;\text{exp}\left(t\Phi \right)=[P_{n}\;\;P_{n-1}\;\;\cdots \;\;P_{2}\;\;P_{1}], \end{array}$$

]where $P_{i}^{\prime }s$ are row vectors of functions of *t*, for *i* = *n*, *n* − 1, ⋯, 1. Then, we get

$$\begin{array}{c} E\left(t\right)=\sum_{i=1}^{n}\int_{0}^{\infty }P_{i}\left(u\right)\lambda \left(t-u\right)S\left(t-u\right)\;\text{exp}\left(-\mu u\right)\;\;\text{d}u. \end{array}$$

If we put

$$\begin{array}{c} E_{i}\left(t\right)=\int_{0}^{\infty }P_{i}\left(u\right)\lambda \left(t-u\right)S\left(t-u\right)\;\text{exp}\left(-\mu u\right)\;\;\text{d}u, \end{array}$$

for *i* = *n*, *n* − 1, ⋯, 1, we could get

$$\begin{array}{cc} \frac{\;\text{d}E_{n}\left(t\right)}{\;\text{d}t} & =\lambda \left(t\right)S\left(t\right)-\left(\zeta_{n}+\mu \right)E_{n}\left(t\right),\\ \frac{\;\text{d}E_{i}\left(t\right)}{\;\text{d}t} & ={\bar{p}}_{i}\zeta_{i+1}E_{i+1}\left(t\right)-\left(\zeta_{i}+\mu \right)E_{i}\left(t\right),\;i=n-1,\cdots ,2,1, \end{array}$$

and this yields

$$\frac{\;\text{d}I_{p}\left(t\right)}{\;\text{d}t}=\zeta_{1}E_{1}\left(t\right)+\sum_{i=2}^{n}p_{i-1}\zeta_{i}E_{i}\left(t\right)-\left(\gamma +\mu \right)I\left(t\right).$$

Applying a similar process to *L*(*t*) yields the Coxian distributed model from (1):

$$\begin{array}{c} \begin{array}{cc} \frac{\text{d}S(t)}{\text{d}t} & =\mu -\lambda \left(t\right)S\left(t\right)+\left(1-\left(1-\kappa \right)\xi \right)\gamma I_{p}\left(t\right)+\gamma_{r}I_{r}\left(t\right)-\mu S\left(t\right),\\ \frac{\;\text{d}E_{n}\left(t\right)}{\;\text{d}t} & =\lambda \left(t\right)S\left(t\right)-\left(\zeta_{n}+\mu \right)E_{n}\left(t\right),\\ \frac{\;\text{d}E_{i}\left(t\right)}{\;\text{d}t} & ={\bar{p}}_{i}\zeta_{i+1}E_{i+1}\left(t\right)-\left(\zeta_{i}+\mu \right)E_{i}\left(t\right),\;i=n-1,\cdots ,2,1,\\ \frac{\text{d}I_{p}\left(t\right)}{\text{d}t} & =\sum_{i=1}^{n}p_{i-1}\zeta_{i}E_{i}\left(t\right)-\left(\gamma +\mu \right)I_{p}\left(t\right),\\ \frac{\;\text{d}L_{m}\left(t\right)}{\;\text{d}t} & =\left(1-\kappa \right)\xi \gamma I_{p}\left(t\right)-\left(\eta_{m}+\mu \right)L_{m}\left(t\right),\\ \frac{\;\text{d}L_{j}\left(t\right)}{\;\text{d}t} & ={\bar{q}}_{j}\eta_{j+1}L_{j+1}\left(t\right)-\left(\eta_{j}+\mu \right)L_{j}\left(t\right),\;j=m-1,\cdots ,2,1,\\ \frac{\text{d}I_{r}\left(t\right)}{\text{d}t} & =\sum_{j=1}^{m}q_{j-1}\eta_{j}L_{j}\left(t\right)-\left(\gamma_{r}+\mu \right)I_{r}\left(t\right),\\ \frac{\text{d}S_{M}\left(t\right)}{\text{d}t} & =\mu_{M}-\lambda_{M}\left(t\right)S_{M}\left(t\right)-\mu_{M}S_{M}\left(t\right),\\ \frac{\text{d}E_{M}\left(t\right)}{\text{d}t} & =\lambda_{M}\left(t\right)S_{M}\left(t\right)-\left(\epsilon_{M}+\mu_{M}\right)E_{M}\left(t\right),\\ \frac{\text{d}I_{M}\left(t\right)}{\text{d}t} & =\epsilon_{M}E_{M}\left(t\right)-\mu_{M}I_{M}\left(t\right), \end{array}\; \end{array}$$ (1′)

where $E\left(t\right)=\sum_{i=1}^{n}E_{i}\left(t\right)$, $L\left(t\right)=\sum_{j=1}^{m}L_{j}\left(t\right)$ and *p*_0_ = *q*_0_ = 1.

In many cases, the threshold principle of in a constant environment is dealt with by using Diekmann-Heesterbeek-Metz(DHM) definition [27] of the basic reproduction number, $R_{0}$. However, it is not applicable to the formulation of threshold principles for population growth in a periodic environment [28]. In particular, the definition of $R_{0}$ in a periodic environment was given by Bacaër and Guernaoui [29], which can be interpreted as the asymptotic per generation. Motivated by the work, H. Inaba presented a new definition for the basic reproduction number as the spectral radius of the generation evolution operator (GEO) [28]. Moreover, it was shown that the supercritical condition $R_{0}>1$ implies existence of positive periodic solution [30]. These facts together yield the existence of a periodic solution to our model that is positive (1′) at which the reproduction number, according to the GEO definition, is bigger than unity.

#### Estimating model parameters

As the Coxian chains in the model (1′) cannot be biologically interpreted because of phenomena that are going out of the absorption state without going through the all the chains [31], we estimate model parameters using two procedures as follows:

(A) Fit survival functions *P* and *Q* to the IP and TTFR data, respectively.
(B) Fit other model parameters *p*_*H*_, *p*_*M*_ and *ξ* in the model (1′) to the parameter values related to *P* and *Q* which are obtained in (A).

More detailed descriptions are as follows: First, we construct the IP and TTFR data as depicted in Fig 1 as an empirical cumulative distribution function (ECDF) and fit these to Coxian CDF (1 − *P*) and (1 − *Q*) in (4). That is, we want to find *P* and *Q* to minimize

$$\begin{array}{cc} \text{SSR} & =\sum_{i=1}^{n_{data}}{\left[\text{EE}\left(t_{i}\right)-\text{ED}\left(t_{i}\right)\right]}^{2}, \end{array}$$ (5)

where EE(*t*_*i*_) is the CDF corresponding to the Coxian distribution and ED(*t*_*i*_) is the empirical cumulative distribution function of IP (TTFR) at the *i*-th sampling time. Especially, *n*_*data*_ = 20 for IP data although 229 individuals were identified because, IP data points were read off the histogram plots in Fig 1 and converted into ECDF data. The difficulty with using the Coxian distribution lies in correctly choosing the number of phases. We use Akaike Information Criterion (AIC), which provides the relative quality of the statistical model for a given data set, for checking the fitting quantity between the empirical data and the distributions and chooses values for *n* and *m* that minimize AIC [32]. As the small sample sizes are small in our case, we use the second-order AIC, AIC_*c*_ which is defined as

$$\begin{array}{c} {\text{AIC}}_{c}≔n_{data}\times \text{log}\left(\text{SSR}/n_{data}\right)+\frac{2K(K+1)}{n_{data}-K-1}, \end{array}$$

where *K* is the number of model parameters and *n*_*data*_ is the sample size [33]. Using lsqnonlin in Matlab, we choose the number of phases of Coxian distributions as *n* = 20 and *m* = 12.

To estimating *p*_*H*_, *p*_*M*_ and *ξ*, we performed a fit of averaged weekly cases for each of the 4 weeks to the incidence data for 4 weeks using our model. Using lsqnonlin in Matlab, we found the values of the parameters that minimize the following objective functional:

$$\begin{array}{c} \begin{array}{cc} J & =\sum_{i=1}^{6}[45603\times \int_{52\times 19+4\times (i-1)+20}^{52\times 19+4\times i+20}(\sum_{k=1}^{n}p_{k-1}\zeta_{k}E_{i}\left(u\right)+\sum_{l=1}^{m}q_{l-1}\eta_{l}L_{l}\left(u\right))\;\text{d}u\\ & \;\;\;\;\;\;\;\;\;\;\;-U_{r}\times \sum_{t=4\times (i-1)+21}^{t=4\times i+20}\text{CD}\left(t\right){]}^{2}, \end{array} \end{array}$$ (6)

where CD(*t*) is the cumulative case data on the *t*-th week and *U*_*r*_ a parameter related to under-reporting. The population of Yeoncheon-gun is 45,603.

### The seasonal reproduction number

The seasonal (or effective) reproduction number $R_{s}$—an alternative form of the DHM definition of the basic reproduction number—is used when when parameters in the model are seasonally periodic [34]. The method of calculation of $R_{s}$ is as follows: $R_{s}$ is determined by the spectral radius of the next-generation operator. Moreover, the next generation operator is given by the following 2 × 2 block matrix in the case of a single vector-borne diseases:

$$\begin{array}{c} {}[\begin{array}{cc} K_{MM} & K_{MH}\\ K_{HM} & K_{HH} \end{array}], \end{array}$$

where *K*_*AB*_ represents the number of infected cases in *B* generated by a single *A* during its infectious period [27]. Obviously *K*_*MM*_ = *K*_*HH*_ = 0 in the case of malaria, where *M* stands for a mosquito and *H* a human, as homogeneous transmission does not exist in this case.

Motivated by [31], we derive the $R_{s}$ as follows:

$$\begin{array}{cc} R_{s}\left(t\right) & =\sqrt{K_{HM}K_{MH}},\\ & =\sqrt{\frac{b^{2}p_{M}p_{H}\rho (t)\epsilon_{M}}{\mu_{M}\left(\epsilon_{M}+\mu_{M}\right)}\{\frac{1-\mu \hat{P}}{\gamma +\mu }+\frac{\left(1-\kappa \right)\xi \gamma \left(1-\mu \hat{P}\right)\left(1-\mu \hat{Q}\right)}{\left(\gamma +\mu \right)\left(\gamma_{r}+\mu \right)}\}}, \end{array}$$

where

$$\begin{array}{c} \begin{array}{cc} K_{HM} & =bp_{M}\rho \{\underset{\text{from}\;\text{primary}\;\text{infection}}{\underset{︸}{\left(1-\mu \hat{P}\right)\cdot \frac{1}{\gamma +\mu }}}+\underset{\text{from}\;\text{relapse}\;\text{due}\;\text{to}\;\text{dormant}\;\text{hypnozoite}}{\underset{︸}{\left(1-\mu \hat{P}\right)\cdot \frac{\gamma }{\gamma +\mu }\cdot \left(1-\kappa \right)\xi \cdot \left(1-\mu \hat{Q}\right)\cdot \frac{1}{\gamma_{r}+\mu }}}\},\\ K_{MH} & =bp_{H}\frac{1}{\mu_{M}}\cdot \frac{\epsilon_{M}}{\epsilon_{M}+\mu_{M}}, \end{array} \end{array}$$ (7)

and

$$\begin{array}{c} \hat{P}=\int_{0}^{\infty }\;\text{exp}\left(-\mu u\right)P\left(u\right)\;\text{d}u,\;\hat{Q}=\int_{0}^{\infty }\;\text{exp}\left(-\mu u\right)Q\left(u\right)\;\text{d}u. \end{array}$$

Note the probability of surviving the exposed class is $1-\mu \hat{P}$ and $1-\mu \hat{Q}$ represents the probability of survival in the class *L*. On one hand, $\hat{P}$ and $\hat{Q}$ are solvable using integration by parts, yielding

$$\begin{array}{c} 1-\mu \hat{P}=\left[Lf_{X}\right]\left(\mu \right)\;\;\;\;\text{and}\;\;\;\;1-\mu \hat{Q}=\left[Lf_{Y}\right]\left(\mu \right). \end{array}$$

Thus, $R_{s}$ is calculated explicitly by

$$\begin{array}{c} \begin{array}{cc} R_{s}=R_{s}\left(t\right) & =\sqrt{\frac{b^{2}p_{M}p_{H}\rho (t)}{\mu_{M}\left(\epsilon_{M}+\mu_{M}\right)\left(\gamma +\mu \right)}\cdot [\sum_{i=0}^{n-1}\{a_{i}\prod_{k=1}^{n-i}\frac{\zeta_{n+1-k}}{\zeta_{n+1-k}+\mu }\}]}\\ & \;\;\;\times \sqrt{1+\frac{(1-\kappa )\xi \gamma }{\left(\gamma_{r}+\mu \right)}[\sum_{j=0}^{m-1}\{b_{j}\prod_{z=1}^{m-j}\frac{\delta_{m+1-z}}{\delta_{m+1-z}+\mu }\}]}, \end{array} \end{array}$$ (8)

where $a_{i}=p_{i}\prod_{j=1}^{n-1-i}{\bar{p}}_{j+i}$ and $b_{i}=q_{i}\prod_{j=1}^{m-1-i}{\bar{q}}_{j+i}$.

Motivated by [35], we consider the proportion of the contribution of relapse in the seasonal reproduction number, $R_{s}$. Using (7), we can reformulate $R_{s}$ as the square root of the sum of the primary-based part, $R_{p}$, and the relapse-based part, $R_{r}$:

$$\begin{array}{cc} R_{s} & =\sqrt{R_{p}+R_{r}}, \end{array}$$

where

$$\begin{array}{cc} R_{p} & =K_{MH}\cdot bp_{M}\rho \left(t\right)\cdot \left(1-\mu \hat{P}\right)\cdot \frac{1}{\gamma +\mu },\\ R_{r} & =K_{MH}\cdot bp_{M}\rho \left(t\right)[\left(1-\mu \hat{P}\right)\cdot \frac{\gamma }{\gamma +\mu }\cdot \left(1-\kappa \right)\xi \cdot \left(1-\mu \hat{Q}\right)\cdot \frac{1}{\gamma_{r}+\mu }]. \end{array}$$

Since $R_{s}>1$ holds if and only if $R_{p}+R_{r}>1$, we can consider the percentage of the contribution of relapse in $R_{s}$, $R_{\%}$ to be

$$\begin{array}{c} \begin{array}{cc} R_{\%} & =\frac{R_{r}}{R_{p}+R_{r}}\times 100\left(\%\right),\\ & =\frac{\gamma \cdot \left(1-\kappa \right)\xi \cdot \left(1-\mu \hat{Q}\right)}{\gamma_{r}+\mu +\gamma \cdot \left(1-\kappa \right)\xi \cdot \left(1-\mu \hat{Q}\right)}\times 100\left(\%\right). \end{array} \end{array}$$ (9)

## Results

### Parameters estimation

**Fig 3:**
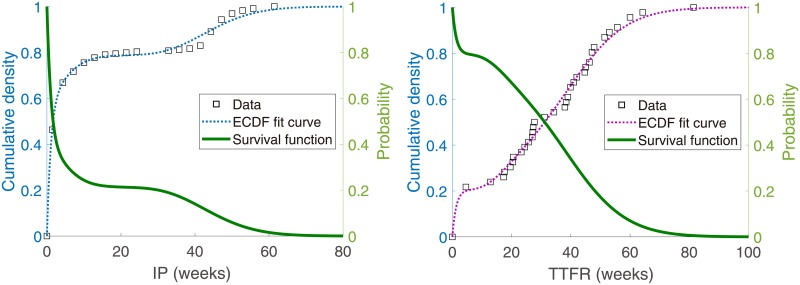
Approximating the empirical cumulative distribution function (ECDF) of IP to a 20-chained Coxian distribution (left), and that of TTFR to a 12-chained Coxian distribution (right). Solid lines represent survival functions in each of the figures. Note that both fitted curves express multimodality.

Parameters consisting of *P* and *Q* and fitted to ECDFs of IP and TTFR to minimize (5), are given in Table 3. Fitted curves agree well with the data, as shown in Fig 3, and satisfactorily express multimodal phenomena well.

**Table 3:** Estimated parameters for Coxian distributed IP and TTFR.

Index (*i*)	IP		TTFR		Index (*i*)	IP		TTFR	
	*ζ*_*i*_	${\bar{p}}_{i}$	*η*_*i*_	${\bar{q}}_{i}$		*ζ*_*i*_	${\bar{p}}_{i}$	*η*_*i*_	${\bar{q}}_{i}$
20	0.835				10	0.433	1.000	0.298	1.000
19	0.455	0.337			9	0.433	1.000	0.298	1.000
18	0.455	0.999			8	0.433	1.000	0.298	1.000
17	0.455	0.999			7	0.433	1.000	0.298	1.000
16	0.436	0.635			6	0.433	1.000	0.298	0.696
15	0.434	0.996			5	0.433	1.000	0.215	1.000
14	0.434	1.000			4	0.434	1.000	0.215	1.000
13	0.434	1.000			3	0.434	1.000	0.215	1.000
12	0.434	1.000	0.773		2	0.434	1.000	0.215	1.000
11	0.434	1.000	0.298	0.794	1	0.434	1.000	0.215	1.000

**Fig 4:**
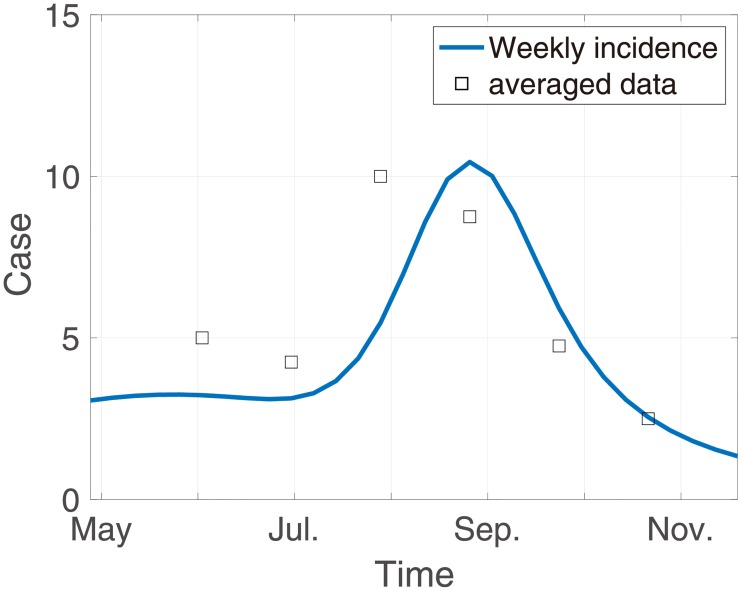
Fitted model results. The solid blue line indicates the model results representing weekly incidence with best fitted parameters and the dots are averaged over the data of weekly cases for each of the 4 weeks.

Next, the model parameters are set as follows: we set the force of mortality of a human as *μ*, with the assumption that the death age of of death an individual is distributed exponentially, so that 1/*μ* is the average life expectancy. We followed concepts of mosquito physiological parameters, such as *μ*_*M*_ and *ρ*(*t*), as discussed in [17]. These parameters were fitted to the observed 2007 mosquito population data in Paju-si and Cheolwon-gun, near Yeoncheon-gun, with exponential and Gaussian assumptions. Since the period of recovery for humans is about 2 weeks [17], we assume 1/*γ* = 2 = 1/*γ*_*r*_. We also set 1/*ϵ*_*M*_ = 7/9 because the mean incubation period for mosquitoes is 9 days [36]. The biting rate of a mosquito with respect to a human, *b*, is assumed to be the average number of bites for that mosquito per week multiplied by the associated Human Blood Index (HBI), which is defined to be by the proportion of the blood in a mosquito population obtained from human [37]. We set *b* = 0.138 with the HBI is assumed to be in the range [0.01, 0.1] and the gonotrophic cycle of mosquito is assumed as about 2.5 days following [17, 38]. The optimal values of the parameters that minimize (6) are *p*_*H*_ = 0.643, *p*_*M*_ = 0.492, *ξ* = 0.133 and *U*_*r*_ = 2.21 and the curve fits with the observed data are as shown in Fig 4.

Detailed values of parameters are listed in Table 4.

**Table 4:** Collected and estimated model parameters.

Parameter	Value (dimension)	Reference	Parameter	Value (dimension)	Reference
*μ*	2.3 × 10^−4^(/week)	[39]	*μ*_*M*_	0.7949 (/week)	Fit in [17]
1/*γ*	2 (weeks)	[17]	1/*γ*_*r*_	2 (weeks)	[17]
*ξ*	0.133	Estimated	*κ*	[0, 1]	
1/*ϵ*_*M*_	9/7 (weeks)	[36]	*b*	0.138 (/week)	[38]
*p*_*H*_	0.643	Estimated	*p*_*M*_	0.492	Estimated
*M*(*t*)	$\;\text{exp}[-{\left(\frac{\;\text{mod}\;(t,52)-31.26}{3.328}\right)}^{2}]/\;\text{exp}[-{\left(\frac{13.26}{3.328}\right)}^{2}]+10^{4}$				Fit in [17]
*ρ*(*t*)	*M*(*t*)/10^4^				Fit in [17]

### Effectiveness of relapse control

#### Contribution of relapse in the seasonal reproduction number

**Fig 5:**
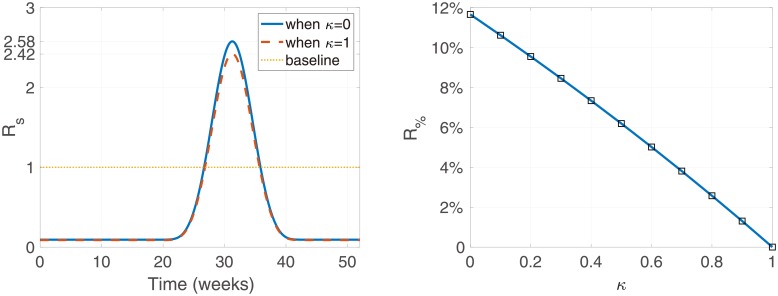
Seasonal reproduction number against time (left), and the percentage of the contribution of relapse in the seasonal reproduction number, $R_{\%}$, against *κ* (right). In the left panel, the blue solid line records $R_{s}$ in the absence of any control (*κ* = 0) and red dashed line records $R_{s}$ in the presence of complete control (*κ* = 1).

In (9), $R_{\%}$ does not depend on seasonality, and is constant. The left panel in Fig 5 depicts the values of the seasonal reproduction number with changing time. The maximum value occurs in summer between July and August, when its value is 2.58 in the absence of relapse control, and 2.42 in the presence of relapse control with complete coverage, respectively. The right panel shows the percentage of the contribution of relapse in the seasonal reproduction number, $R_{s}$, in the presence of a relapse treatment with coverage rate *κ*. It is shown that relapse treatment reduces the contribution proportion of relapse. With the current coverage of relapse while *Primaquine* is being used, the model shows a maximum relapse contribution proportion of 11.7% in Korea. However, if the coverage of the relapse treatment were 90%, it is possible to reduce $R_{\%}$ to 1.3%.

#### Malaria prevalence

**Fig 6:**
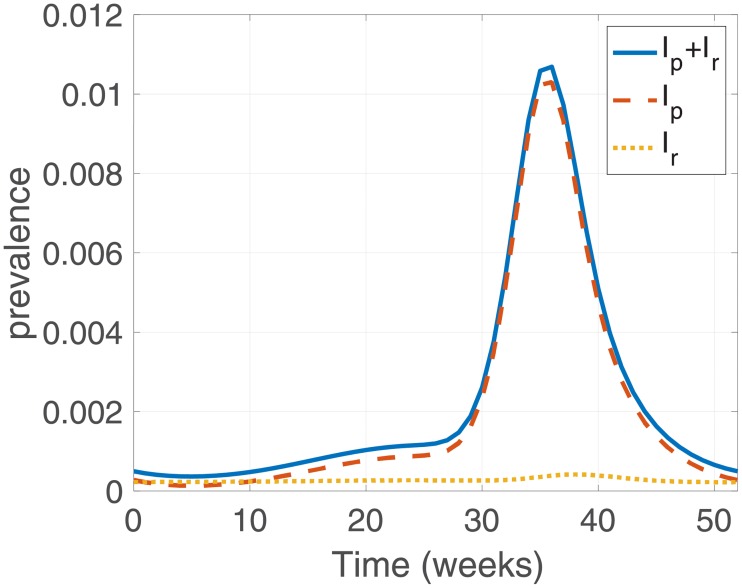
Model result of the percentage of infectious humans with the current level of control. The blue solid line shows the total number of infectious humans, *I*_*p*_ + *I*_*r*_, the red dashed line indicates primary infected humans, *I*_*p*_, and the yellow dotted line indicates infectious individuals who have relapsed, *I*_*r*_.

Fig 6 illustrates the results of the malaria prevalence model using the parameters estimated in the previous section. During a year, 11.8% of cases resulted from relapse in total prevalence. Overall, primary infection is more prevalent than relapsed infection, but in winter, infected humans who have relapsed are more common. While total prevalence of malaria in humans tends to be similar to the prevalence of primary infections, relapsed prevalence remains more or less constant throughout the year, besides having a peak during summer, just like the case of primary infections. This is because relapsed infections are less affected by changes in the mosquito population than primary infections.

**Fig 7:**
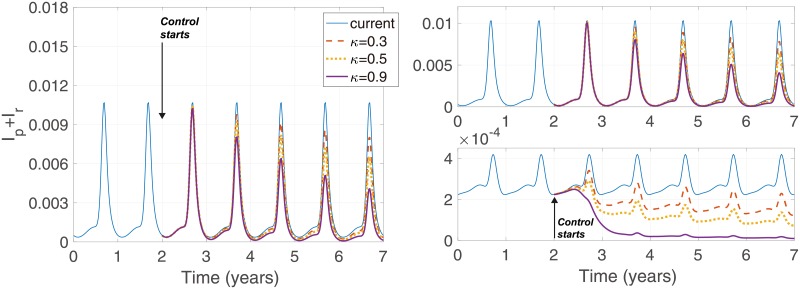
Model results of *I*_*p*_ + *I*_*r*_, *I*_*p*_, and *I*_*r*_ with control. Control starts at 2 and remains constant for 5 years with a rate *κ*.

Fig 7 depicts the effect of constant control over 5 years. The gradual weakening of the scale of outbreaks over several consecutive years suggests the possibility of complete elimination of the parasites. 90% coverage of relapse also reduces relapsed prevalence to zero within 5 years. A further finding is that control not only reduces the relapse prevalence but also reduces the rate of primary infections. The upper right panel of the figure shows that the peak of prevalence of primary infections reduces by more than 2 times after 5 years of relapse treatment with 90% coverage.

#### Eradication of malaria

**Fig 8:**
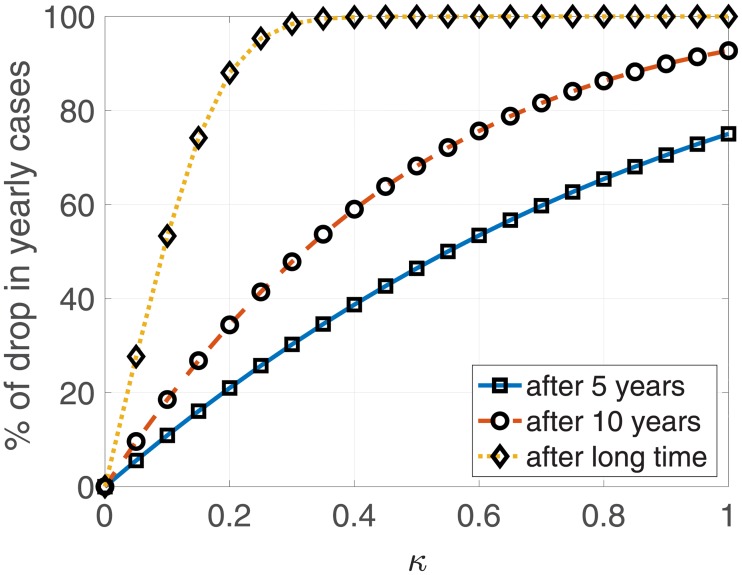
The percentage of drops in yearly cases after control.

Fig 8 summarizes the results related to the percentages of reduction in yearly cases after application of control that could explain the eradication of malaria. Theoretically, control at an application rate of 20% for a very long time can reduce cases by more than 90%, and can exterminate malaria at an application rate of approximately 40%. However, in the case of low coverage control over a period of 5 years or 10 years, control can reduce a great proportion of malaria incidences, but extinction is not possible. This means that malaria extinction is impossible via short-term intensive control.

#### Potential effect of manipulated behavior of mosquito caused by malaria parasites

Recent studies have shown that manipulated behaviors caused by malaria parasites in mosquitoes can increase the force of infection of malaria by more than twofold [40, 41]. These behavioral changes are caused by a survival instinct in malaria parasites, and therefore they tend to intensify as malaria is controlled [42]. Hence, the absence of these manipulation phenomena in the usual scenario can be an obstacle to identify appropriate intervention schedules for vector-borne disease control efforts [43].

**Fig 9:**
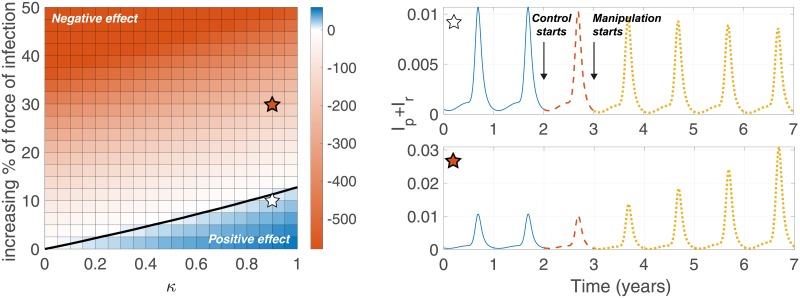
The effectiveness of relapse control considering manipulated behavior of mosquitoes. Left colormap shows the percentages of reduction in yearly cases during 5-year control against *κ* and an increasing rate in the force of infection. A positive value indicates that the case is decreasing through control, and a negative value means the opposite. In the left panel, we assume that manipulation occurs one year after control starts. The black solid line shows the contour that the percentage of drops is 0. The upper and lower figures on right show the percentages of infectiousness when *κ* = 0.9 and when there is a 10%, 30% increase in the force of infection in humans with one year delay in manipulation after the beginning of control methods, respectively.

We describe the potential effect of this feeding behavioral change over long periods of control in Fig 9. The solid line in the left panel shows that the bifurcation between the percentages of reduction in yearly cases with a 5-year control is positive and negative. Although a high rate of control has been implemented, it shows that a slight increase in the force of infection caused by control methods can have a negative manipulative effect without being significantly affected by the delay at the start of the manipulation.

## Discussion

We used a transmission model to characterize the relapse of malaria considering realistic intrinsic distributions of incubation periods and the time to first relapse. We used Coxian distributed IP and TTFR distribution to model bimodality of incubation and relapse, which are distinct from other countries. Thus, the performance of our suggested method for statistical inferring of relapse related parameters from prevalence data is lower when considering with other countries. To the best of our knowledge, there has been no prior modeling study considering heuristic time to first relapse data for malaria dynamics in Korea. Moreover, incubation time and realistic relapse time distributions are considered more than others in the existing literature (refer to the S1 Appendix).

The seasonal reproduction number was used to calculate the proportion of contribution of relapse, indicating the effect of relapse on the epidemiology of malaria in the Republic of Korea. By dividing the number by two parts, primary infection based part and relapse based part, we could get the proportion explicitly. Next, we performed the study to demonstrate the effectiveness of anti-relapse treatment on the assumption that relapse treatments with greater coverage compared to the present could be used. The treatment was assumed to have a higher coverage rate than in the present, and we conducted the study under the assumption that the treatment rate was *κ* at the current rate. The results show that the control of relapse reduces total prevalence of malaria by reducing the numbers of both relapsed and primary infected individuals even though relapsed cases are significantly fewer in number than primary infected cases. We also studied whether malaria eradication is possible through relapse treatment. Theoretically, eradication can be achieved with a relapse coverage rate of 40% after a very long time, but eradication of malaria within 10 years is not possible even if the relapse is controlled at a very high rate. Therefore, further developments in vector control or prevention strategies are necessary. Moreover, considering the manipulated behavior of mosquitoes, our results demonstrate that negative effects on control might be yielded even if a high rate of treatment is implemented. We did not consider drug resistance in this study. If this were considered, a strategy for eradication of malaria in the near future might be necessary, which is difficult to control by merely controlling relapse. Therefore, it is proposed that mass treatment using new drugs should be performed with gradual increase in vector control considering the mosquitoes’ feeding behavioral changes. The study will also help disease prevention authorities in implementing timely and effective malaria control measures when new malaria drugs become commercially available.

Our work has several limitations. We used a data fitting method to quantify the current level of relapse treatment with absence of time series relapsed data and time to first relapse data for each patient. In addition, population of malaria mosquitoes in the Republic of Korea have a bimodal form that rapidly increases around May, temporarily decreases during the rainy season and then increases rapidly after the rainy season ends. As such, the bimodality of the number of patients shown in Fig 4 is affected by the number of mosquitoes. However, in this model, the influence of the peak around May is designed only by relapse and incubation. This reason may cause overestimation of total relapse prevalence in this model although about 5% of estimated cases of official relapse in 2018 were reported in the Republic of Korea [7]. If further data are investigated, suggested method may be applied to the current management of malaria in Korea by investigating the specific contents that can be applied centering on policy application.

## Conclusion

In this work, we constructed a mathematical model to quantify the relapse of malaria in Korea and to evaluate the effectiveness of control methods in reducing relapse. Our model was able to express the distribution of multimodal incubation periods and the time to first relapse in continental climate zones where seasonality is evident. In this study, it was found that control of malaria relapse in Korea and the progression of control under the current modes of vector control can reduce malaria in the short-term but can have potentially negative effects in the long-term. The results suggest that control at higher vector control levels may result in reduction of malaria.

A number of recommendations for further research may be provided. For example, considering the monthly differences in the intrinsic distribution, the model generated by the age structured PDE may be reconstructed to exhibit the temporal effect on malaria prevalence. In addition, combinations with higher vector control will allow the reconstruction of the model to determine the appropriate timings and strategies for malaria control.

## Supporting information

S1 Appendix Comparison of distributions fit with ones in existing literatures. (PDF)
