# Supplementary material for: A mathematical model for assessing the effectiveness of controlling relapse in Plasmodium vivax malaria endemic in the Republic of Korea
Source: PLoS One. 2020 Jan 24;15(1):e0227919. doi: 10.1371/journal.pone.0227919 (PMC6980521; doi:10.1371/journal.pone.0227919)
Supplement: S1 Appendix — (PDF) [file pone.0227919.s001.pdf]

S1 Appendix: Comparison of distributions fit with ones in existing literatures

In this section, we compare fitting results of the fitted distributions with incubation period (IP) data and the time to first relapse (TTFR) data, respectively. In nonparametric tests, the Kolmogorov–Smirnov metric are mostly used to compare a sample with a reference probability distribution [1]. The test uses the test statistics as the supremum of distances pointwise between empirical CDF and a reference CDF. However, if the distribution of data are shown as bimodal phenomena, the Wasserstein metric, which is defined as

$$W_1(\text{ECDF}, F_{reference}) = \int_0^1 \left|\text{ECDF}^{-1}(t) - F^{-1}_{reference}(t)\right| \, \mathrm{d}t,$$

where $\text{ECDF}(t)$ is empirical CDF and $F_{reference}$ is a reference CDF, is more appropriate to compare with two distributions [2].

Use the Wasserstein metric, we compare the 4 types of distributions, which are used in the existing literatures, for the incubation period (IP) data and 2 types of distributions for the time to first relapse (TTFR) data as the follows:

(A) Exponential distribution with the mean $1/a$ [3–5] :

$$P_E(t) = \exp(-at).$$

(B) Gamma distribution with the shape parameter a and the scale parameter b [4] :

$$P_G(t) = \frac{1}{b^a \Gamma(a)} \int_0^t x^{a-1} \exp\left(-\frac{x}{b}\right) \mathrm{d}x.$$

(C) Bi-exponential distribution with means $1/a$ and $1/b$, respectively [6]:

$$P_{BE}(t) = \varphi \exp(-at) + (1-\varphi)\exp(-bt) \text{ where } 0 \le \varphi \le 1.$$

(D) Joint distribution between Exponential and Dirac-delta [7] :

$$P_{ED}(t) = \begin{cases} \varphi \exp(-at) + (1-\varphi) & t \in [0, \tau] \\ \varphi \exp(-at) & t \in (\tau, \infty) \end{cases} \text{ where } 0 \le \varphi \le 1.$$

We use $\varphi = 0.80$, $a = 0.274$ and $b = 0.021$ for P_{BE} and P_{ED} of the IP data [8] and others are fitted with MATLAB `distribution fitter`. The Empirical CDF and fit cumulative distribution functions are depicted in the **Fig A**, and the Wasserstein metric between ECDF and fit CDF are in the **Table A**. The results are shown that our model is much closer to the empirical distribution in the sense of the Wasserstein metrics, than other models.

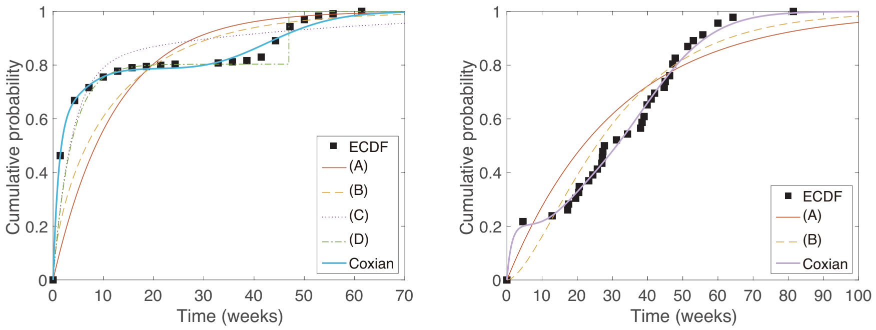

Fig A. Fitting the empirical cumulative distribution function (ECDF) of IP (left), and that of TTFR (right) to various distributions.

Table A. Wasserstein metric between ECDF and the fit CDF.

	Type (A)	Type (B)	Type (C)	Type (D)	Coxian
IP data	6.53×10^{-2}	5.26×10^{-2}	6.45×10^{-2}	1.63×10^{-2}	1.04×10^{-2}
TTFR data	9.52×10^{-2}	6.23×10^{-2}			1.49×10^{-2}

References

1. In: Kolmogorov–Smirnov Test. New York, NY: Springer New York; 2008. p. 283–287. Available from: https://doi.org/10.1007/978-0-387-32833-1_214.

2. Masson C. Detecting Anomalies Using Statistical Distances; 2018. Available from: https://www.youtube.com/watch?v=U7xdiGc7IRU/.

3. Chamchod F, Beier JC. Modeling Plasmodium vivax: relapses, treatment, seasonality, and G6PD deficiency. Journal of theoretical biology. 2013;316:25–34.

4. Roy M, Bouma MJ, Ionides EL, Dhiman RC, Pascual M. The potential elimination of Plasmodium vivax malaria by relapse treatment: insights from a transmission model and surveillance data from NW India. PLoS Neglected Tropical Diseases. 2013;7(1):e1979.

5. Kim JE, Choi Y, Lee CH. Effects of climate change on Plasmodium vivax malaria transmission dynamics: A mathematical modeling approach. Applied Mathematics and Computation. 2019;347:616–630.

6. Nah K, Kim Y, Lee JM. The dilution effect of the domestic animal population on the transmission of P. vivax malaria. Journal of Theoretical Biology. 2010;266(2):299–306.

7. Nah K, Nakata Y, Röst G. Malaria dynamics with long incubation period in hosts. Computers & Mathematics with Applications. 2014;68(9):915–930.

8. Kim SJ, Kim SH, Jo SN, Gwack J, Youn SK, Jang JY. The long and short incubation periods of Plasmodium vivax malaria in Korea: the characteristics and relating factors. Infection & Chemotherapy. 2013;45(2):184–193.
